# Inferring Phylogenetic Networks from Gene Order Data

**DOI:** 10.1155/2013/503193

**Published:** 2013-08-28

**Authors:** Alexey Anatolievich Morozov, Yuri Pavlovich Galachyants, Yelena Valentinovna Likhoshway

**Affiliations:** Limnological Institute of the Siberian Branch of the Russian Academy of Sciences, 3 Ulan-Batorskaya Street, Irkutsk 664033, Russia

## Abstract

Existing algorithms allow us to infer phylogenetic networks from sequences (DNA, protein or binary), sets of trees, and distance matrices, but there are no methods to build them using the gene order data as an input. Here we describe several methods to build split networks from the gene order data, perform simulation studies, and use our methods for analyzing and interpreting different real gene order datasets. All proposed methods are based on intermediate data, which can be generated from genome structures under study and used as an input for network construction algorithms. Three intermediates are used: set of jackknife trees, distance matrix, and binary encoding. According to simulations and case studies, the best intermediates are jackknife trees and distance matrix (when used with Neighbor-Net algorithm). Binary encoding can also be useful, but only when the methods mentioned above cannot be used.

## 1. Introduction

Gene order data gain increasing popularity in the phylogenetic community because of several advantages they have, compared with gene sequences. First, in most cases the genome structure evolves slower than DNA or protein sequence, allowing the inference about ancient events with less noise level [1]. Second, like all phylogenomic studies, the analysis of genomic rearrangements is not hampered by conflicts between gene trees and species tree. One can expect the rearrangements-based inference and phylogenomics in general to become more and more widespread as DNA sequencing cost continues to decline and new computational tools are developed to deal with this kind of data.

As with any analysis, it is based upon several assumptions. First, every genome is represented as a permutation of homologous markers, which are usually directed. All genomes in the dataset should contain the same set of markers. Though in the majority of studies these markers are genes (hereinafter referred to as “genes”), they can be contiguous parts of a chromosome of any reasonable length. Such a permutation is traditionally represented as a sequence of signed numbers with absolute values being identifiers of elements and a sign denoting direction. Second, a set of operations on a permutation is limited to some subset of all actually possible evolutionary events. Inversions are the most common, but there can be also translocations, double-cut-and-join operations, and several other events [1].

In the majority of phylogenetic studies, the evolutionary relationships of taxa are represented by phylogenetic trees. Despite their usefulness for biology, a phylogenetic tree by definition is able to display only one divergence-based scenario. Real evolution, on the other hand, is not limited to diverging taxa: recombination and horizontal gene transfer occur in all major groups of organisms. Even in cases when a tree-like evolution is safe to assume, sometimes it is useful to simultaneously visualize all conflicting scenarios supported by the dataset [2]. Reticulate evolutionary events are currently beyond the scope of the gene order analysis because a similar set of genes in all genomes is assumed. On the other hand, ambiguous data do not often allow the choice of a single tree. To solve these problems, *phylogenetic networks*, that is, nontree graphs describing evolutionary relationships, were proposed. Many types of phylogenetic networks exist [2], but in this study we are interested only in *split networks* [3].

An idea of the split network is based upon one crucial observation: every branch of the phylogenetic tree defines a split or bifurcation of taxa set, that is, separates it into two parts. Split is called trivial if one of its parts contains only one taxon. Such a split corresponds to a leaf branch on the phylogenetic tree. A set of splits is called compatible if it can be represented by an unrooted phylogenetic tree. Phylogenetic inference in this framework is reduced to generating a set of nontrivial splits and representing them as a graph.

A split network is a generalization of mathematical concept of a phylogenetic tree. It is able to represent both compatible and incompatible split sets. Unlike unrooted tree, a split network may use several parallel edges to represent any given split. Deletion of all edges corresponding to a split divides the network in exactly two connected components, one containing all taxa from one part of the split, and another containing taxa from the other. The edge lengths are defined by split weight, which can be of different sense depending on the algorithm used to generate a split set [2].

## 2. Materials and Methods

### 2.1. Building Phylogenetic Networks

The main idea of this work is to apply existing network-building algorithms to intermediate data, which were generated according to the structure of studied genomes. We used a set of phylogenetic trees (consensus network algorithm), inversion distance matrix (split decomposition and Neighbor-Net algorithms), and binary encoding (parsimony-splits algorithm) as intermediate data. In all cases, we considered rearrangements in unichromosomal genomes consisting of directed markers and limited the evolutionary process to inversions. The latter assumption is not applied to binary encoding (see details below).

We did not filter the splits with any of the algorithms and intermediates. The aim of the additional filtering is to obtain a relatively simple set of splits (e.g., cyclic or weakly compatible). Such a set of splits corresponds to a network with simple topology, which can be easily drawn on a flat surface. However, such a simplification causes removal of some splits from the resulting network, which potentially leads to loss of important data. We decided to sacrifice simplicity of the network for the sake of its accuracy.

The data transformations, jackknife analysis, and simulations were done by custom Perl scripts, which are available upon request. All network construction algorithms are implemented in SplitsTree4 software by Huson and Bryant [4]. Computations were done at Irkutsk High-Performance Computer Center (ISDCT SB RAS, Irkutsk).

### 2.2. Jackknife Trees

Since the function of split networks is to represent conflicting trees, the most obvious solution is to generate a bunch of trees and sum them up into a network. To obtain a set of trees, we conducted a jackknife procedure: 40% of genes were chosen randomly and removed from all permutations, preserving the order of the remaining ones. We generated 100 replicates and built phylogenetic trees using COGNAC package by Kang et al. [5].

Phylogenetic network was built by consensus network algorithm [6]. This algorithm uses all splits which are present in all input trees to build a network. The algorithm was set up to include a split into the network if it is present in at least 10% of the input trees. In this case, the split weights and therefore the edge lengths in the resulting network are equal to a jackknife support of the corresponding bifurcation, that is, a proportion of input trees that include this bifurcation.

### 2.3. Distance Matrix

Distance matrix is a common kind of intermediate data which allows building phylogenetic trees and networks from different raw data using the same algorithms. GRAPPA package [7] was used to generate the matrix of pair-wise inversion distances. This distance metric designates minimum number of inversion operations necessary to transform an initial gene permutation into a target one. Two algorithms were applied to distance matrices: Neighbor-Net [8] and split decomposition [2].

### 2.4. Binary Encoding

Binary encoding (BE) represents a set of permutations as a matrix of binary characters [9]. The rows of this matrix correspond to permutations. Columns are the pairs of genes in all four relative orientations (+N +M, +M +N, +N −M, and −N +M). Every element of matrix equals 1 if two genes are adjacent in genome in given directions; otherwise, it is equal to 0. Such an approach allows us to promptly analyze large datasets. It can use existing software and does not make explicit assumption about the nature of rearrangement process. BE matrices were processed by parsimony-splits algorithm [10].

The median network algorithm is also popular in the analysis of binary sequences. However, a crucial step of the analysis is to reconstruct ancestral states. In case of BE, the algorithm does not account for actual evolution of underlying gene order, therefore the reconstruction of ancestral sequences will be incorrect. Thus, we did not use this approach in our work.

### 2.5. Simulations

For simulations, we generated Yule trees for 10 and 20 taxa with the branch lengths sampled from Poisson distribution. The expected branch lengths *λ* ranged from 1 to 10 inversions. For each combination of the taxa amount and *λ*, we generated 100 random trees. The gene orders evolved according to topology of these trees by random inversions starting from the identity permutation of 100 genes at the root of the tree. Permutations observed at the tree leaves were used as an input for methods described above.

The resulting networks were compared with the true underlying tree. We assessed only a network topology, that is, a set of splits. We counted nontrivial splits that are either present in the true tree, but not in the network (false negative (FN)), or *vice versa* (false positive (FP)). Obviously, all trivial splits are always present in both tree and network, therefore they were excluded from the analysis.

Two values were obtained from the FN and FP counts to compare performance of the methods. First value is sensitivity, that is, probability for any split in the true tree to be included into the phylogenetic network. Another value is a positive predictive value (PPV), which has an opposite meaning: probability that a network split belongs to the true tree. We calculated neither specificity nor negative predictive value because the true negative count, which is the amount of all possible splits on given taxa set minus the amount of splits in the true tree, is always several orders of magnitude larger than that of FN and FP.

Split weights were not taken into account because their meaning is different in the network-building approaches used. This is an important flaw of the simulation procedure because once a split is present in the network, it will increase sensitivity (or decrease PPV) of the method, even if the weight of this split is vanishingly small.

### 2.6. Case Studies

We used two real datasets in the case studies. The first is Campanulaceae dataset, which is commonly used to evaluate performance of the rearrangement-based phylogenetic inference software [9, 11, 12], provided as test data with the Badger package [12]. This dataset is poorly resolved: all trees in the aforementioned papers contain multifurcations and, in some cases, support values are not provided (see Figure 3(e) for example).

Another dataset consists of six chloroplast genomes of diatoms and two genomes of diatom-derived chloroplasts of dinoflagellates. All these are circular genomes 120–130 kbp long, containing from 154 to 159 genes, including tRNAs. Each chloroplast DNA bears a long inverted repeat that contains genes for rRNAs and several proteins. Phylogenetic relationships of taxa are quite well established [13], and our tree inferred from the order of genes in chloroplast genomes supports the conventional scenario (Figure 2(e)).

We removed one of the copies of inverted repeat from all diatom genomes, thus transforming sequences from circular to linear. Then all common genes were assigned numbers and marked with signs depending on their orientation. The resulting dataset consisted of eight permutations of 149 genes each. Both real datasets were analyzed in the same way as the simulated data.

## 3. Results and Discussion

### 3.1. Simulations

**Figure 1:**
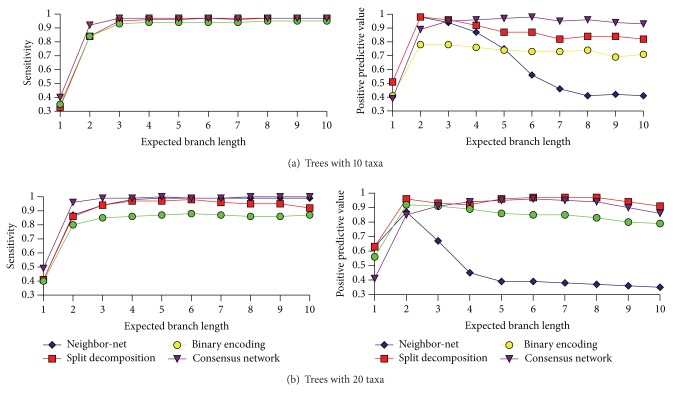
Simulation results.

According to simulation studies (Figure 1), the analysis of a set of the jackknife trees with the consensus network algorithm appeared to be the best way to build split networks. Networks generated by this method had the highest sensitivity and PPV in most tests. However, the reconstruction of a set of trees is significantly more CPU-intensive than computation of either distance matrix or binary encoding.

The split decomposition algorithm is slightly outperformed by the consensus network approach in terms of both sensitivity and PPV. The distance matrix can be computed significantly faster than a set of trees. It took less than a minute on a desktop computer for all tests performed. Additionally, this method guarantees to produce a weakly compatible set of splits, ensuring less complicated network.

PPV of Neighbor-Net algorithm significantly decreases with increasing lengths of tree branches. On the other hand, sensitivity is similar to that of other methods.

The parsimony-splits algorithm applied to the binary encoding is different from other methods. It does not analyze permutations, but processes binary matrices built from them. This may be useful, if the evolutionary process is not assumed to be based mainly on inversions. However, in our “inversions-only” simulations, the networks built with this method have the lowest sensitivity and the second lowest PPV.

### 3.2. Case Studies

Obviously, one can never know exactly which tree is actually true for a real dataset. Therefore, by analyzing real data: one can only assess relative complexity of a network, that is, how many splits are included, and whether it is congruent with the trees obtained on the same dataset with other methods.

**Figure 2:**
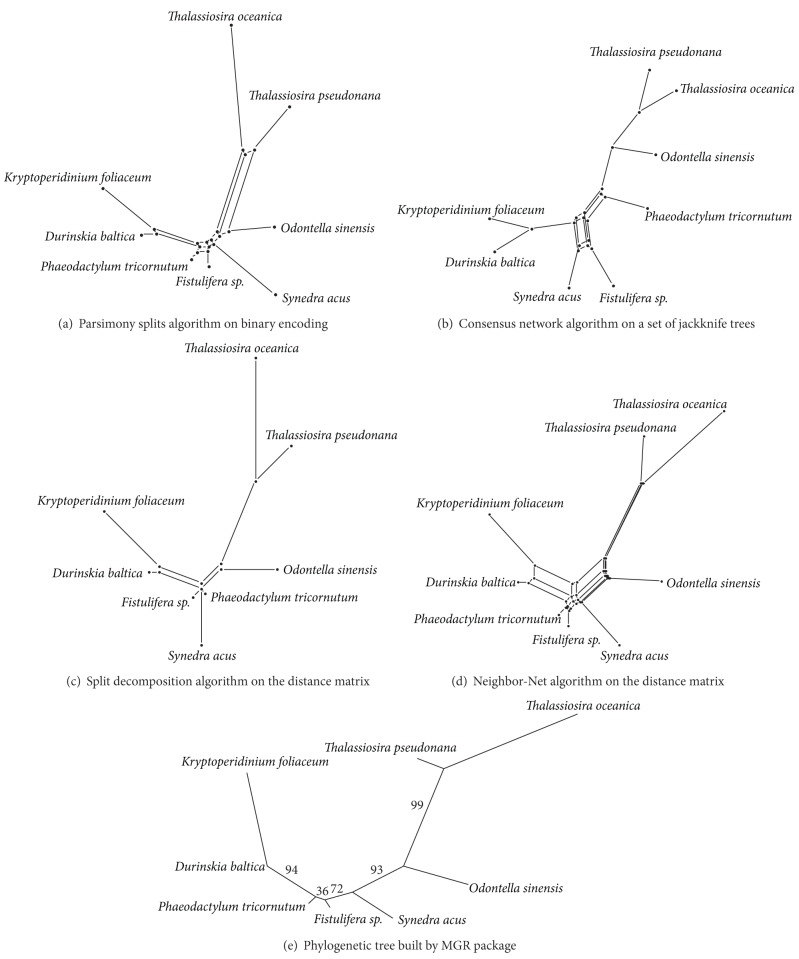
Split networks and reference tree for the Bacillariophyta dataset.

On Bacillariophyta dataset, we first built a phylogenetic tree using MGR package (Figure 2(e)) [11]. This tree is congruent to the trees obtained using molecular phylogenetic analysis of several diatom genes [13]. Therefore, we used topology of this tree as a reference to assess quality of split networks.

The Neighbor-Net algorithm has produced the most complex network (Figure 2(d)) with the largest number of splits. Most of these splits, however, have low weights, making the best scenario clearly visible. Binary encoding-based network (Figure 2(a)) is smaller in terms of splits. Its topology is also the closest to the MGR tree. Two other networks contain significant flaws. Consensus network gives the highest support to positions of *Synedra*, *Phaeodactylum*, and *Fistulifera* that contradict our MGR tree (Figure 2(b)). It is also the only network that contains a 3-dimensional structure, which makes it much harder to read. Network built by split decomposition algorithm has a small number of additional splits, but it also lacks a few crucial ones, leaving the *Synedra*/*Phaeodactylum*/*Fistulifera* relationships completely unresolved (Figure 2(c)).

**Figure 3:**
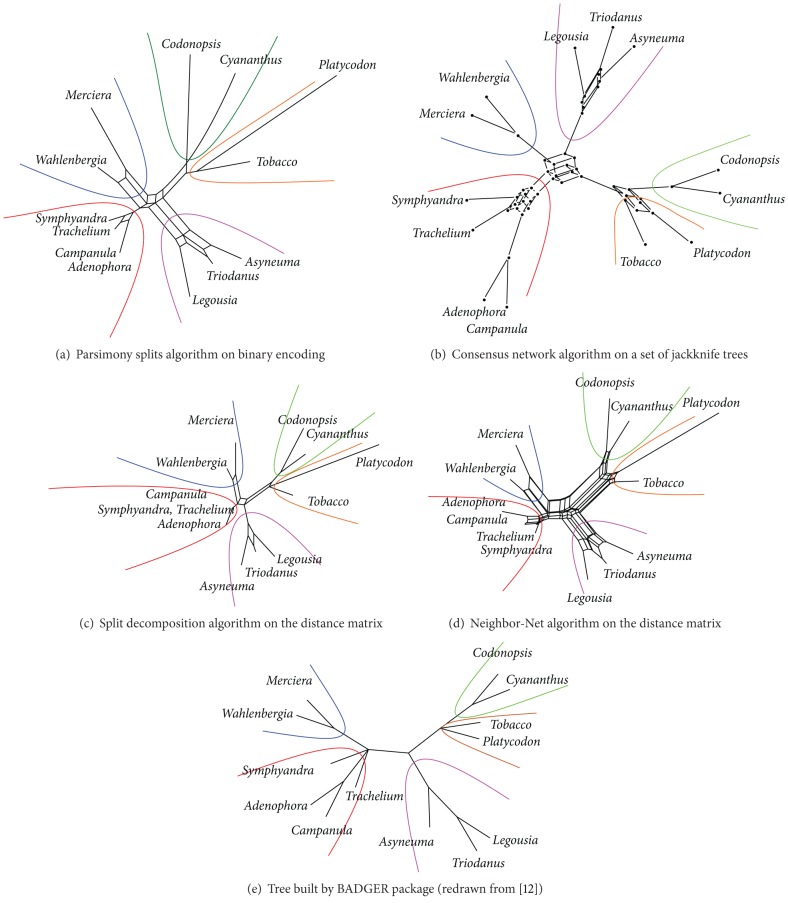
Split networks and reference tree for the Campanulaceae dataset.

Campanulaceae dataset was confirmed to be ambiguous. All methods support five relatively well-supported monophyletic clusters (marked by colors in Figure 3), but neither positions of taxa inside them nor relations of the clusters are resolved. The same conflict is present in consensus trees built with other algorithms ([10, 12, 13], see Figure 3(e)). Networks clearly show that some groups can be reliably separated from each other, yet the complete reconstruction cannot be done based solely on this dataset.

## 4. Conclusions

In this study, we propose several methods to build split networks using the gene order data via generating the intermediate datasets. We used a set of jackknife trees, an inversion distance matrix, and a binary encoding of the gene order as intermediate data. The performance of these methods is shown to vary depending on input data. Furthermore, the suggested methods are different in assumptions and mathematical approaches behind them. Below we summarize *pro et contra* of every method.

A set of jackknife trees is useful in most cases. In simulations, it performs well in terms of both sensitivity and positive predictive value. Moreover, it produces networks with bifurcation support as a split weight, which is very useful when comparing the reliability of different scenarios. Since, in the absence of additional split filtering, consensus network approach does not limit the produced split set to weakly compatible or circular, it can create networks of very complex, hard to read topology.

Networks are computationally cheaper to build with the distance matrix as an intermediate dataset. This matrix can be analyzed by Neighbor-Net and split decomposition algorithms. When comparing these two algorithms, it is necessary to take into account that PPV is much less important than sensitivity. If the data clearly support only one scenario, it would not be obscured by addition of several low-weight splits represented by barely visible edges. On the other hand, if several contradictory trees are supported, a resulting network must include splits from all of them. In this case, PPV will decrease. However, the use of a network instead of a tree is aimed at representing this contradiction.

Unlike split decomposition algorithm which generates multifurcations, Neighbor-Net tends to add a lot of low-weight splits into network. Moreover, it has slightly higher sensitivity. These two features seem to be advantageous to apply the Neighbor-Net approach. However, the split network generated via Neighbor-Net, which is always producing a circular set of splits, may lack some splits versus the network derived with split decomposition algorithm. For detailed example, see archaeal chaperonins dataset [8]. This problem only appears for very contradictory scenarios, so in the majority of cases Neighbor-Net is preferable.

Analysis of binary encoded genome structures by parsimony-splits algorithm has lower sensitivity and PPV than the rest of methods. Still, it can be useful for very large datasets, when other approaches are computationally expensive. The fact that evolutionary process is not assumed to consist of some limited set of operations is also advantageous when no such set can be proposed. However, in most cases, the consensus network or Neighbor-Net approach would be more reliable.
